# Supplementary material for: Infrared molecular fingerprinting of blood-based liquid biopsies for the detection of cancer
Source: eLife. 2021 Oct 26;10:e68758. doi: 10.7554/eLife.68758 (PMC8547961; doi:10.7554/eLife.68758)
Supplement: Figure 2—source data 2. [file elife-68758-fig2-data2.zip › Figure 2-source data 4/Readme.docx]

Supplementary Data 1

Description for the proper application of the trained machine learning models provided within Supplementary Data 1

In the framework of this study, classification models were trained to answer several clinical questions. The trained models of the most relevant classifications (see also Table 1) are included in this Supplementary Data 1 and are intended for future use in a follow-up study or for studies of other groups. It is important to note that the provided final models were trained on the data of the entire cohorts (see Supplementary Table 2). Therefore, there is no internal test set.

This document’s main purpose is to provide the reader with the technical details for using the trained classification models provided within Supplementary Data 1. Before applying the models, it must be ensured that the input data have the same structure and should also be pre-processed in the same way as the data used for model training. The entire procedure was performed in Python (v. 3.7.6) in the machine-learning framework of Scikit-Learn (v. 0.23.2).

## Data pre-processing

In this first section, we outline a detailed description of each step of the pre-processing pipeline.

#### Raw data

The data was imported directly from the exports of the measurement device. An example of a recorded spectrum is presented in the figure bellow:


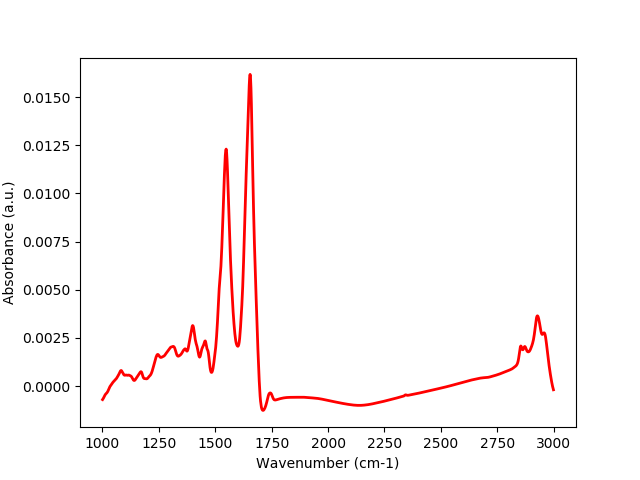


The spectrum is truncated form wavenumbers 1000.88098145 cm^-1^ to 2998.78588867. The spectral resolution corresponds to half a cm^-1^ (more precisely 0.5190437223776289 cm^-1^) per point. In case the input spectrum has a different frequency axis, an interpolation to the above grid must be performed.

#### Water correction

The first pre-processing step, after importing the raw data, is the water correction. Negative absorption, which occurs if the liquid sample contains less water than the reference (pure water), was corrected as follows. We first take measurements of pure water to assess the absorption as function of the wavenumber. The data of the water measurement are plotted below and can be found in the file *water_spectrum.txt*:


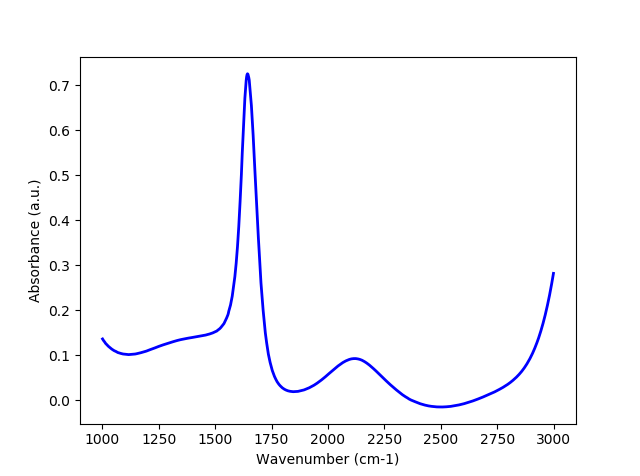


It is known from measurements of dried serum or plasma, that there is no significant absorption in the wavenumber region 2000-2300 cm^-1^, resulting in a flat absorption baseline. We used this fact as a criterion for adding to each spectrum a measured water absorption spectrum to account for the missing water in the sample measurement and minimize the average slope in this region in order to obtain a flat baseline. The minimization is done by running repeated simulations of adding the water spectrum to the sample $k$ times, for different values of $k$ and finding the optimal value that minimizes the absolute value of the first derivative on average within the region 2000-2300 cm^-1^. The first derivative is calculated using the Savitzky-Golay filter as follows from the SciPy module using the following parameters:

scipy.signal.savgol_filter(x, window_length=9, polyorder=2, deriv=1, delta=1.0, axis=- 1, mode='interp', cval=0.0)

The resulting optimal values for $k$ are in the range between 0.003-0.005. The resulting spectrum is shown below:


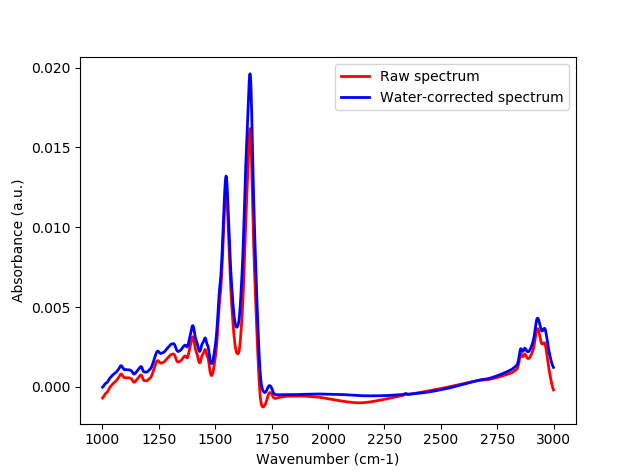


#### Removal of silent region

After the water correction, all spectra were truncated to exclude the ‘silent region’ (region in which there are no major or dominant absorption peaks of molecules found in serum or plasma.), between 1850-2800 cm^-1^. The truncation was implemented by finding the wavenumbers closest to the above limits, using the following function:

def find_nearest(array, value):
 array = np.asarray(array)
 idx = (np.abs(array - value)).argmin()
 return array[idx]

#### Normalization

Finally, all spectra were normalized, using Euclidean (L2) norm. The implementation was done using the function *normalize* of the Scikit-Learn module as follows:

sklearn.preprocessing.normalize(X, norm='l2')

The resulting spectrum is shown below:


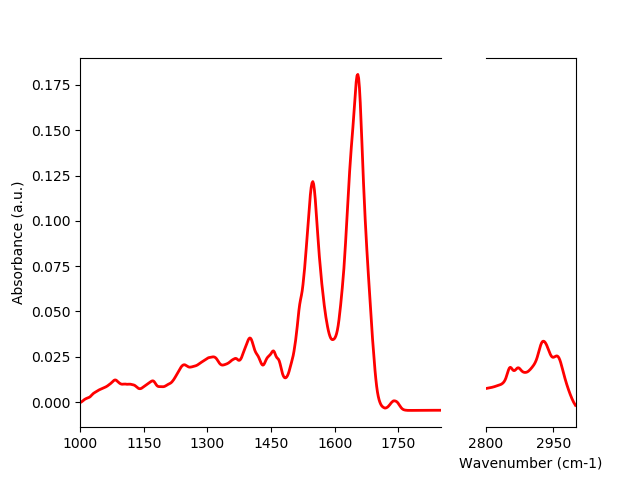


## Classification models

The 7 binary classification models contained within Supplementary Data 1 correspond to the 7 clinical questions that were addressed in Fig. 2 and Table 1:

- LuCa vs. NSR
- LuCa vs. SR
- LuCa vs. MR
- BrCa vs. NSR
- BlCa vs. NSR
- PrCa vs. NSR
- PrCa vs. SR
- PrCa vs. MR

An understanding of the definitions of the questions and the characteristics of the cohorts (as defined in the main manuscript) is a prerequisite for the correct use of the models. These final models were trained using linear SVM, as implemented in Scikit-Learn:

sklearn.svm.SVC(kernel="linear", probability=True, C=1)

#### Import

The models can be imported using the *joblib* module, as follows

from joblib import load
clf = load('filename.joblib')

#### Predictions and ROC AUC

SVM-based models require the data to be centred and scaled, i.e. $(X-\overline{X})/\sigma$. This is taken care automatically using the *StandardScaler* function of Scikit-Learn, as follows:

from sklearn.preprocessing import StandardScaler

st_sc = StandardScaler()
st_sc.fit(X)
X = st_sc.transform(X)

After this, the model can be used to make predictions on new instances included in a NumPy array$X$, as follows

clf.predict(X)

The value of the ROC AUC can be computed on the new data using the following piece of code:

from sklearn.metrics import roc_curve

scores = clf.decision_function(X)
fpr, tpr, _ = roc_curve(target, scores)
roc_auc = auc(fpr, tpr)

The variable *target* corresponds to a list with the class labels (ground truths), if known.

Important note: While models trained using a specific version of Scikit-Learn might load in another, operations performed on such data could give different and unexpected results.
