# Supplementary material for: Infrared molecular fingerprinting of blood-based liquid biopsies for the detection of cancer
Source: eLife. 2021 Oct 26;10:e68758. doi: 10.7554/eLife.68758 (PMC8547961; doi:10.7554/eLife.68758)
Supplement: Transparent reporting form [file elife-68758-transrepform1.docx]

***eLife’s* transparent reporting form**

We encourage authors to provide detailed information *within their submission* to facilitate the interpretation and replication of experiments. Authors can upload supporting documentation to indicate the use of appropriate reporting guidelines for health-related research (see [EQUATOR Network](http://www.equator-network.org/%20)), life science research (see the [BioSharing Information Resource](https://biosharing.org/)), or the [ARRIVE guidelines](http://www.plosbiology.org/article/info:doi/10.1371/journal.pbio.1000412) for reporting work involving animal research. Where applicable, authors should refer to any relevant reporting standards documents in this form.

If you have any questions, please consult our Journal Policies and/or contact us: [editorial@elifesciences.org](mailto:editorial@elifesciences.org).

**Sample-size estimation**

- You should state whether an appropriate sample size was computed when the study was being designed
- You should state the statistical method of sample size computation and any required assumptions
- If no explicit power analysis was used, you should describe how you decided what sample (replicate) size (number) to use

Please outline where this information can be found within the submission (e.g., sections or figure legends), or explain why this information doesn’t apply to your submission:

A statistical power calculation for the sample size was performed prior to the study and is included in the study protocol. The main findings of the sample size calculation are given in the Materials and Methods section within the Study design subsection.

**Replicates**

- You should report how often each experiment was performed
- You should include a definition of biological versus technical replication
- The data obtained should be provided and sufficient information should be provided to indicate the number of independent biological and/or technical replicates
- If you encountered any outliers, you should describe how these were handled
- Criteria for exclusion/inclusion of data should be clearly stated
- High-throughput sequence data should be uploaded before submission, with a private link for reviewers provided (these are available from both GEO and ArrayExpress)

Please outline where this information can be found within the submission (e.g., sections or figure legends), or explain why this information doesn’t apply to your submission:

No replicates were measured. However, quality control serum samples were measured in each measurement run to evaluate the measurement error (see Methods). We found that the measurement error was very small compared to the biological variability of the samples. In addition, the results obtained on a subset from plasma and serum samples from the same individuals were similar, indicating that no technical variance or device variation affected the measurement results.

**Statistical reporting**

- Statistical analysis methods should be described and justified
- Raw data should be presented in figures whenever informative to do so (typically when N per group is less than 10)
- For each experiment, you should identify the statistical tests used, exact values of N, definitions of center, methods of multiple test correction, and dispersion and precision measures (e.g., mean, median, SD, SEM, confidence intervals; and, for the major substantive results, a measure of effect size (e.g., Pearson's r, Cohen's d)
- Report exact p-values wherever possible alongside the summary statistics and 95% confidence intervals. These should be reported for all key questions and not only when the p-value is less than 0.05.

Please outline where this information can be found within the submission (e.g., sections or figure legends), or explain why this information doesn’t apply to your submission:

There is a statistical analysis subsection within the Materials and Methods section describing the statistical analysis in detail. In addition, all necessary details (e.g., exact values if N) of the analysis are given in the Figure legends and the corresponding Tables in the Supplementary information.

(For large datasets, or papers with a very large number of statistical tests, you may upload a single table file with tests, Ns, etc., with reference to sections in the manuscript.)

**Group allocation**

- Indicate how samples were allocated into experimental groups (in the case of clinical studies, please specify allocation to treatment method); if randomization was used, please also state if restricted randomization was applied
- Indicate if masking was used during group allocation, data collection and/or data analysis

Please outline where this information can be found within the submission (e.g., sections or figure legends), or explain why this information doesn’t apply to your submission:

The samples were mostly measured in the order in which they arrived at the measurement site. As sample collection and delivery is to some extent a stochastic process (both cases and references were collected over the entire period), no additional randomisation of the measurement order was performed. This information is given in the Materials and Methods section.

**Additional data files (“source data”)**

- We encourage you to upload relevant additional data files, such as numerical data that are represented as a graph in a figure, or as a summary table
- Where provided, these should be in the most useful format, and they can be uploaded as “Source data” files linked to a main figure or table
- Include model definition files including the full list of parameters used
- Include code used for data analysis (e.g., R, MatLab)
- Avoid stating that data files are “available upon request”

Please indicate the figures or tables for which source data files have been provided:

The datasets generated and analysed during the current study will not be published publicly due to privacy regulations under the General Data Protection Regulation (EU) 2016/679. Anonymized raw datasets are available for review purposes. The raw data includes clinical data from patients, including textual clinical notes and contain information that could compromise subjects’ privacy or consent. Any additional information and data are available upon reasonable request. The trained machine learning models are provided within Supplementary Data 1 along with basic Python code for importing them in a python script. The custom code used for the production of the results presented in this manuscript is stored in a persistent repository at the Leibniz Supercomputing Center of the Bavarian Academy of Sciences and Humanities (LRZ), located in Garching, Germany. The code can only be shared upon reasonable request, as its correct use depends heavily on the settings of the experimental setup and the measuring device and should therefore be clarified with the authors.
